# Bacteremic community-acquired pneumonia due to *Klebsiella pneumoniae*: Clinical and microbiological characteristics in Taiwan, 2001-2008

**DOI:** 10.1186/1471-2334-10-307

**Published:** 2010-10-25

**Authors:** Yi-Tsung Lin, Yuan-Yu Jeng, Te-Li Chen, Chang-Phone Fung

**Affiliations:** 1 Division of Infectious Diseases, Department of Medicine, Taipei Veterans General Hospital, Taipei, Taiwan; 2 Institute of Clinical Medicine, School of Medicine, National Yang-Ming University, Taipei, Taiwan

## Abstract

**Background:**

*Klebsiella pneumoniae *is the major cause of community-acquired pyogenic infections in Taiwan. This retrospective study evaluated the clinical and microbiological characteristics of bacteremic community-acquired pneumonia due to *K. pneumoniae *in Taiwanese adults.

**Methods:**

The clinical characteristics of bacteremic community-acquired pneumonia (CAP) in adults due to *K. pneumoniae *were compared to those of adults with bacteremic CAP due to *Streptococcus pneumoniae *at a tertiary medical center in Taiwan from 2001-2008. Risk factors for mortality of bacteremic CAP due to *K. pneumoniae *were analyzed. All clinical isolates of *K. pneumoniae *were examined for capsular serotypes, hypermucoviscosity phenotype, aerobactin and *rmpA *gene.

**Results:**

*K. pneumoniae *was the dominant cause of bacteremic CAP and was associated with a more fulminant course and a worse prognosis than bacteremic CAP due to *Streptococcus pneumoniae*. Initial presentation with septic shock and respiratory failure were independent risk factors for both early and total mortality. Serotype K1 and K2 comprised around half of all isolates. There were no significant differences in the clinical characteristics of patients with bacteremic CAP due to K1/K2 and non-K1/K2 isolates. Hypermucoviscosity phenotype as well as the aerobactin and *rmpA *genes were highly prevalent in the *K. pneumoniae *isolates.

**Conclusions:**

*K. pneumoniae *continued to be the dominant cause of bacteremic CAP in Taiwanese adults during 2001-2008. Initial presentation with septic shock and respiratory failure were independent risk factors for both early and total mortality from *K. pneumoniae *bacteremic CAP. Serotypes K1/K2 comprised around half of all isolates, but did not predispose patients to a poor clinical outcome. Physicians should be aware of the poor prognosis of any patient with bacteremic *K. pneumoniae *CAP and monitor these patients more closely.

## Background

*Klebsiella pneumoniae *is the major cause of liver abscess, Gram-negative bacillary meningitis, brain abscess, lung abscess, thoracic empyema, prostatic abscess, deep neck infection and complicated skin and soft tissue infections in Taiwan [1], and comprised 4.8% of the common causative pathogens of community-acquired pneumonia (CAP) in a multi-center survey [2]. Although *K. pneumoniae *is generally considered an infrequent cause of CAP in the United States and Europe, a large prospective study showed that *K. pneumoniae *was not only a frequent etiology of severe CAP, but also an independent risk factor for mortality [3].

A number of studies have suggested that bacteremia is a risk factor for death in patients with CAP [4-7]. Although previous studies investigated the characteristics and treatment strategy for bacteremic pneumococcoal pneumonia, few studies of bacteremic CAP due to *K. pneumoniae *have been reported and most of these were conducted before 2000 [8-10]. In one global study, pneumonia accounted for 29% and 62% of all cases of community-acquired *K. pneumoniae *bacteremia in Taiwan and South Africa, respectively; however, only four cases of community-acquired bacteremic *K. pneumoniae *pneumonia were seen in the United States, Argentina, Europe, or Australia [10].

The present study compared the clinical characteristics of bacteremic CAP due to *K. pneumoniae *with the characteristics of bacteremic CAP due to *Streptococcus pneumoniae *and the virulence factors of invasive *K pneumoniae *isolates in a tertiary medical center over an 8-year period in Taiwan.

## Methods

### Patient selection

Microbiology records were used to identify consecutive patients with bacteremic CAP admitted between January 2001 and December 2008 at Taipei Veterans General Hospital, a 2,900-bed tertiary medical center in northern Taiwan. The medical records of patients with bacteremic CAP due to either *K. pneumoniae *or *S. pneumoniae *were reviewed. CAP was defined as an acute lower respiratory tract infection characterized by the following: (1) an acute pulmonary infiltrate evident on CXR and compatible with pneumonia, (2) confirmatory findings of the clinical examination, and (3) acquisition of the infection outside a hospital, long-term care facility, or nursing home [11]. Bacteremic CAP was defined as the presence of at least one blood culture sampled within 48 h of presentation in the hospital, yielding a pathogen presumed to be the cause of the pneumonia [11], and the absence of other infectious sources which could account for the bacteremia. Patients younger than 18 years old were excluded. The diagnosis was reconfirmed by 2 infectious diseases specialists. The institutional review board of the Taipei Veterans General Hospital approved this study.

### Data collection and definition

The following data were collected for each patient: demographic characteristics, underlying diseases or risk factors, clinical variables present at admission, Acute Physiologic and Chronic Health Evaluation (APACHE) II score, pneumonia severity index (PSI) score [12], laboratory data, empirical antibiotics received, chest radiograph reports, days of hospitalization, deaths less than 48 hrs after admission, and in-hospital death. Concordant antibiotic therapy was defined as initial antibiotic therapy including any antibiotic to which the infecting organism was sensitive based on the microbiology report. The diagnosis of septic shock was based on a systolic blood pressure of <90 mmHg and peripheral hypoperfusion with clinical or bacteriological evidence of uncontrolled infection. Neurologic diseases encompassed stroke and head injury. Chronic obstructive pulmonary diseases, bronchiectasis, and old pulmonary tuberculosis belonged to the subgroup of chronic lung diseases. The outcome of bacteremic CAP was defined as in-hospital mortality measured at 28 days after onset of bacteremic CAP. Early mortality was defined as death less than 48 hrs after onset of bacteremic CAP.

### Microbiology laboratory procedures

The VITEK 2 system (bioMérieux, Marcy l'Etoile, France) was used to confirm bacterial identifications. The antimicrobial susceptibility of *K. pneumoniae *was tested by the disk diffusion method and interpretations were made according to the guidelines of the Clinical and Laboratory Standards Institute [13].

### Serotyping and detection of hypermucoviscosity phenotype, aerobactin, and *rmpA *gene

All isolates of *K. pneumoniae *causing bacteremic CAP were serotyped by a countercurrent immunoelectrophoresis method as described previously [14]. Antisera were provided by the Gram Negative Serotyping Unit of the Laboratory of Health Care Associated Infection (Health Protection Agency Centre for Infections, London). *K. pneumoniae *ATCC9997 (K2) was used as a control strain. All isolates with K1 and K2 serotypes were confirmed by PCR for serotype-specific targets within the K1 and K2 *cps *clusters as described previously [15]. Using crude genomic DNA as the template, we performed PCRs to amplify the *rmpA *and aerobactin genes as described previously [16]. Strains with the hypermucoviscosity phenotype were defined by a positive string test as described previously [16].

### Statistics

Contingency data were analyzed by 2-tailed chi-square test or Fisher's exact test, and continuous data were analyzed by Student's t test or the Mann-Whitney U test. A *p *value of <0.05 was considered to be statistically significant, and all probabilities were 2-tailed. Variables with *p *values of <0.10 in univariate analyses subsequently entered in the multivariate analysis. The multivariate logistic regression model was used to evaluate the risk factors for mortality in bacteremic CAP caused by *K. pneumoniae*. All statistical analyses were performed with SPSS, version 15.0 for Windows (SPSS).

## Results

### Clinical characteristics of patients with bacteremic CAP due to *K. pneumoniae*

During the study period, 95 patients with bacteremic CAP due to either *K. pneumoniae *or *S. pneumoniae *were identified from 148 consecutive patients with bacteremic CAP. Excluding two patients whose infections were determined to be caused by polymicrobial pathogens mixed with *S. pneumoniae*, the remaining 93 bacteremic pneumonia patients were divided into two groups with *K. pneumoniae *or *S. pneumoniae *as the causative pathogen. During the study period, bacteremic CAP was more frequently caused by *K. pneumoniae *than *S. pneumoniae*. The clinical characteristics of the patients with infections caused by these two pathogens are compared in Table 1. Patients with *K. pneumoniae *infection had a significantly higher mortality rate (55.1% vs. 27.3%, respectively, *P *= 0.007), a significantly higher early mortality rate (36.7% vs. 13.6%, respectively, *P *= 0.011), a significantly higher prevalence of PSI Risk Class V (89.8% vs. 61.4%, respectively, *P *= 0.001), a significantly higher prevalence of ICU admission (57.1% vs. 31.8%, respectively, *P *= 0.014), a significantly higher prevalence of initial presentation with respiratory failure (61.2% vs. 31.8%, respectively, *P *= 0.005), a significantly higher prevalence of APACHE II score > 25 (71.4% vs. 29.5%, respectively, *P *< 0.001) and a greater likelihood of initial presentation with septic shock (51% vs. 31.8%, respectively, *P *= 0.061). None of the 49 cases of bacteremic CAP due to *K. pneumoniae *had concurrent liver abscess or other metastatic infection. All patients with bacteremic *K. pneumoniae *CAP received concordant antibiotic treatment according to the results of susceptibility testing.

**Table 1:** Comparison of clinical characteristics of patients with bacteremic *K. pneumoniae *and *S. pneumoniae *community-acquired pneumonia

Characteristic	*K. pneumoniae* (n = 49) No. (%)	*S. pneumoniae* (n = 44) No. (%)	*p*-value
Age, mean years ± SE	73 ± 14	76 ± 11	0.893
Male sex	39 (79.6)	39 (88.6)	0.236
Predisposing condition or risk factor			
Diabetes mellitus	17 (34.7)	11 (25)	0.309
Malignancy	14 (28.6)	11 (25)	0.698
Neurologic disorders	12 (24.5)	12 (27.3)	0.759
Chronic kidney disease stage≧4	13 (26.5)	13 (29.5)	0.746
Chronic lung diseases	23 (46.9)	21 (47.7)	0.939
Liver cirrhosis	4 ( 8.2)	3 ( 6.8)	1.000
Alcoholism	5 (10.2)	1 ( 2.3)	0.207
Autoimmune disease	3 ( 6.1)	1 ( 2.3)	0.619
No underlying diseases	3 ( 6.1)	5 (11.4)	0.470
APACHE II score > 25	35 (71.4)	13 (29.5)	<0.001
Days of hospitalization	28 ± 24	21 ± 15	0.929
Initial presentation with septic shock	25 (51)	14 (31.8)	0.061
Initial presentation with respiratory failure	30 (61.2)	14 (31.8)	0.005
PSI Risk Class V	44 (89.8)	27 (61.4)	0.001
ICU admission	28 (57.1)	14 (31.8)	0.014
Concurrent lung abscess	3 ( 6.1)	0 ( 0)	0.244
Early mortality (deaths ≦ 48 hrs)	18 (36.7)	6 (13.6)	0.011
In-hospital mortality	27 (55.1)	12 (27.3)	0.007

Values given as mean ± SD or No. of patients (%).APACHE, Acute Physiologic and Chronic Health Evaluation; PSI, Pneumonia Severity Index; ICU, intensive care unit.

### Laboratory findings for bacteremic community-acquired pneumonia due to *K. pneumoniae*

Table 2 compares the chest radiography and laboratory findings between patients with bacteremic *K. pneumoniae *and *S. pneumoniae *CAP. Patients with bacteremic *K. pneumoniae *CAP had a higher prevalence of bilateral involvement on chest radiography (65.3% vs. 22.7%, respectively, *p *< 0.001) and a lower leukocyte count (11.6 ± 6.7 × 103/μL vs. 14.4 ± 7.4 × 103/μL, respectively, *P *= 0.044).

**Table 2:** Comparison of chest radiography and laboratory findings of patients with bacteremic *K. pneumoniae *and *S. pneumoniae *community-acquired pneumonia

Characteristic	*K. pneumoniae* (n = 49) No. (%)	*S. pneumoniae* (n = 44) No. (%)	*p*-value
Chest radiography at admission			
Unilateral involvement	17 (34.7)	34 (77.3)	<0.001
Bilateral involvement	32 (65.3)	10 (22.7)	<0.001
Cavity lesion	3 (6.1)	1 (2.3)	0.619
Initial laboratory value			
Leucocyte count, × 103/μL	11.6 ± 6.7	14.4 ± 7.4	0.044
Platelet, × 103/μL	212 ± 101	196 ± 107	0.969
Albumin, g/dL	2.6 ± 0.6	2.8 ± 0.6	0.072
C-reactive protein, mg/dL	21.8 ± 11.2	24.9 ± 1.5	0.664
Serum creatinine, mg/dL	2.3 ± 1.9	2.3 ± 1.4	0.186
Total biliburin, mg/dL	1.2 ± 2.7	1.1 ± 1.3	0.809
Glucose, mg/dL	220 ± 168	208 ± 202	0.528

Values given as mean ± SD or No. of patients (%).

### Factors associated with mortality in *K. pneumoniae *bacteremic CAP

The results of univariate analyses of variables related to death in bacteremic CAP due to *K. pneumoniae *are listed in Table 3. In the logistic regression analysis, initial presentation with shock (OR 16.9, 95% CI 3.0-94.4, *P *= 0.001) and respiratory failure (OR 13.3, 95% CI 2.3-77.6, *P *= 0.004) were the only independent factors associated with fatal outcome. Early mortality compromised two thirds of deaths in patients with bacteremic CAP due to *K. pneumoniae*. Univariate analyses of variables related to early mortality and subsequent logistic regression analysis revealed that initial presentation with septic shock (OR 18.9, 95% CI 3.0-118.5, *p *= 0.002) and respiratory failure (OR 22.6, 95% CI 2.2-229.9, *P *= 0.008) were as well the only independent factors associated with early mortality.

**Table 3:** Risk factors for mortality in bacteremic CAP caused by *K. pneumoniae*

Characteristic	Survivors (n = 22) No. (%)	Non-survivors (n = 27) No. (%)	*p*-value
Age > 65 years	15 (68.2)	22 (81.5)	0.282
Male	19 (86.4)	20 (74.1)	0.288
Initial presentation with septic shock	4 (18.2)	21 (77.8)	<0.001
Initial presentation with respiratory failure	7 (31.8)	23 (85.2)	<0.001
APACHE II score>25	10 (45.5)	25 (92.6)	<0.001
PSI Risk Class V	18 (81.8)	26 (96.3)	0.160
ICU admission	10 (45.5)	18 (66.7)	0.136
Malignancy	5 (22.7)	9 (33.3)	0.414
Diabetes mellitus	10 (45.5)	7 (25.9)	0.153
Liver cirrhosis	1 ( 4.5)	3 (11.1)	0.617
Chronic kidney disease stage≧4	6 (27.3)	7 (25.9)	0.915
Chronic lung diseases	12 (54.5)	11 (40.7)	0.336
Neurologic disorders	7 (31.8)	5 (18.5)	0.282
Autoimmune disease	1 ( 4.5)	1 ( 3.8)	1.000
Alcoholism	2 ( 9.1)	3 (11.1)	1.000
No underlying diseases	1 ( 4.0)	2 ( 7.4)	0.609
Chest radiography--bilateral involment	14 (63.6)	18 (66.7)	0.825
Leucopenia ^a^	2 (9.1)	4 (14.8)	0.678
Thrombocytopenia	2 (9.1)	9 (33.3)	0.043
C-reactive protein, mg/dL	20.9 ± 12.1	22.7 ± 10.6	0.482

Values given as mean ± SD or No. of patients (%).^a ^Leucopenia was defined as leucocyte count less than 4000 × 103/μL. APACHE, Acute Physiologic and Chronic Health Evaluation; PSI, Pneumonia Severity Index; ICU, intensive care unit.

### Susceptibility testing

All 49 *K. pneumoniae *isolates showed uniform resistance to ampicillin and susceptibility to a number classes of antibiotics including cephalosporins, aminoglycosides, and fluoroquinolones.

### Capsular serotypes and virulence factors of *K. pneumoniae*

Serotypes K1 and K2 accounted for 14.3% (7/49) and 38.8% (19/49) of all *K. pneumoniae *isolates, respectively. The prevalence of non K1/K2 serotypes of 23 isolates revealed K5 (n = 3), K20 (n = 2), and unidentified type (n = 18). There were no significant difference in the clinical characteristics of patients with bacteremic CAP due to K1/K2 and non-K1/K2 isolates (Table 4). All K1/K2 isolates (n = 26) and 20 out of 23 non-K1/K2 isolates were positive for the hypermucoviscosity phenotype, aerobactin and *rmpA *genes. Three other non-K1/K2 isolates were neither hypermucoviscous nor positive for aerobactin or *rmpA *genes. There were no statistically significant differences in the prevalence of the hypermucoviscosity phenotype, the aerobactin and *rmpA *genes between the K1/K2 and non-K1/K2 isolates *P *= 0.096).

**Table 4:** Relation of *K. pneumoniae *serotype to clinical characteristics

Characteristic	Serotype K1, n = 7 No. (%)	Serotype K2, n = 19 No. (%)	Serotype non-K1/K2, n = 23 No. (%)	***p*-value**^**a**^
Age > 65 years	5 (71.4)	14 (73.7)	18 (78.3)	0.674
Male sex	4 (57.1)	15 (78.9)	20 (87)	0.229
Underlying disease or risk factor				
Malignancy	1 (14.3)	5 (26.3)	8 (34.8)	0.365
Diabetes mellitus	5 (71.4)	6 (31.6)	6 (26.1)	0.234
Liver cirrhosis	0 (0)	2 (10.5)	2 (8.7)	1.000
Chronic kidney disease stage≧4	1 (14.3)	5 (26.3)	7 (30.4)	0.560
Chronic lung diseases	2 (28.6)	10 (52.6)	11 (47.8)	0.907
Neurologic disorders	3 (42.9)	2 (10.5)	7 (30.4)	0.363
Autoimmune disease	0 (0)	2 (10.5)	1 (4.3)	1.000
Alcoholism	1 (14.3)	2 (10.5)	2 (8.7)	1.000
CXR--bilateral involvement	6 (85.7)	12 (63.2)	14 (60.9)	0.539
Leucopenia ^b^	1 (14.3)	3 (15.8)	2 (8.7)	0.671
Concurrent lung abscess	0 (0)	2 (10.5)	1 (4.3)	1.000
Initial presentation with septic shock	4 (57.1)	10 (52.6)	11 (47.8)	0.674
Initial presentation with respiratory failure	4 (57.1)	12 (63.2)	14 (60.9)	0.962
APACHE-II score>25	5 (71.4)	15 (78.9)	15 (65.2)	0.365
PSI Risk Class V	7 (100)	17 (89.5)	20 (87)	0.655
ICU admission	3 (42.9)	12 (63.2)	13 (56.5)	0.934
Early deaths ≦ 48 hrs	3 (42.9)	7 (36.8)	8 (34.8)	0.790
In-hospital death	4 (57.1)	11 (57.9)	12 (52.2)	0.698

Values given as No. of patients (%).^a ^Serotype K1/K2 vs. Serotype non-K1/K2.

^b ^Leucopenia was defined as leucocyte count less than 4000 × 103/μL. APACHE, Acute Physiologic and Chronic Health Evaluation; PSI, Pneumonia Severity Index, ICU: intensive care unit.

## Discussion

*K. pneumoniae *is the major cause of pyogenic infections worldwide, especially in Taiwan [1]. The present study further highlights the role of *K. pneumoniae *as a dominant pathogen in bacteremic CAP in Taiwan. A previous investigation of bacteremic CAP in Taiwan from June 1988 to September 1991 found that *K. pneumoniae *was the most frequently isolated sole pathogen (34.1%) [9]. The present study demonstrated that *K. pneumoniae *was the dominant pathogen from cases of bacteremic CAP (33.1%) in a large tertiary-care hospital in Taiwan between 2001 and 2008. Though conducted in a single institute, the findings of the present study are compatible with the previous cohort [9], and again reflect the highly endemic nature of *K. pneumoniae *infections in Taiwan [10,17].

Previous studies in Taiwan found that approximately 40-50% of community-acquired *K. pneumoniae *bacteremia was associated with diabetes mellitus [10,18], and patients with liver cirrhosis were more likely to develop *K. pneumoniae *bacteremia [19]. In addition, bacteremic CAP due to *K. pneumoniae *was significantly associated with alcoholism[10]. However, in the present study, the most common underlying diseases were chronic lung diseases, which may be explained by the older age of the study population. Diabetes, a classic risk factor for community-acquired *K. pneumoniae *infection, did not predispose patients to bacteremic *K. pneumoniae *CAP in this study. This finding suggests that the mechanism responsible for the susceptibility to *K. pneumoniae *infection may be different for bacteremic CAP than for other clinical manifestations, such as liver abscess. Further study is needed to define the risk factors which predispose patients to the development of bacteremic *K. pneumoniae *CAP.

There has been limited study of clinical characteristics of bacteremic *K. pneumoniae *CAP in the last decade. In this study, we found that the severity of CAP and the prevalence of bilateral involvement on chest X-ray were significantly greater in patients with bacteremic *K. pneumoniae *CAP. Patients with bacteremic *K. pneumoniae *CAP also had worse clinical course than patients infected with *S. pneumoniae*. Knowledge of the different characteristics of *K. pneumoniae *and *S. pneumoniae *bacteremic CAP is of great importance for physicians in endemic areas such as Taiwan. It is also vital for the medical community in which physicians are constantly faced with the diagnostic and prognostic challenges of CAP patients with uncommon pathogens. A previous study found patients infected with *K. pneumoniae *had increased frequency of lower platelet count, leucopenia, higher serum albumin, and male sex [8]. The lower leucocyte count of patients with *K. pneumoniae *bacteremic CAP in the present study is consistent with these findings.

A study of ICU cases with bacteremic CAP in South Africa from January 1982 through July 1985 found that mortality rate was 80% in patients with *K. pneumoniae *infections and 67% in patients with *S. pneumoniae *infections [8]. Another study from 1996 through 1997 found that death rates from bacteremic CAP due to *K. pneumoniae *were 54% in Taiwan and 56% in South Africa [10]. Mortality rate from bacteremic CAP due to *K. pneumoniae *remained high (55%) in this study despite advances in antimicrobial agents and supportive care. The independent risk factors for mortality from bacteremic CAP due to *K. pneumoniae *in this study were initial presentation with septic shock and respiratory failure. Previous study found that the first 48 hours of evolution of CAP were critical [20] and early mortality was also high in the present study. We also found that initial presentation with septic shock and respiratory failure were the only factors associated with early mortality. A previous study found that alcoholism was a risk factor for poor outcome of bacteremic CAP due to *K. pneumoniae *[21]. However, the present study did not find any association between specific underlying disease subgroups and mortality due to *K. pneumoniae *bacteremic CAP. Physicians should be aware of the high mortality rate in patients with bacteremic *K. pneumoniae *CAP and monitor patients more closely.

In this study, capsular serotype K1 and K2 comprised 14.3% and 38.8%, respectively, of all *K. pneumoniae *isolates. Previous studies reported that serotypes K1 and K2 were more common in isolates from community-acquired infections in Taiwan, South Africa, and Singapore than from hospital-acquired isolates in these countries or elsewhere [17,22]. Metastatic infection is an important complication of *K. pneumoniae *liver abscess in Taiwan, and certain capsular serotypes of *K. pneumoniae *including K1 and K2 are thought to play an important role in the development of distal metastatic infections, generally being considered the predominant virulent strains [22-24]. Our previous study indicated that serotype K1 and K2 comprised 59% of *K. pneumonaie *in community-acquired thoracic empyema or complicated parapneumonic effusion but did not predispose patients to poor outcome compared with other non-K1/K2 serotypes [25]. In the present study, patients with K1/K2 serotype *K. pneumoniae *isolates also did not have a worse outcome than those with non-K1/K2 serotype of *K. pneumoniae *isolates, and none of the patients with bacteremic CAP due to *K. pneumoniae *developed metastatic infection. This finding suggests that pulmonary infection with *K. pneumoniae *might carry little risk for distant metastasis even in patients with bloodstream infection. It also suggests that though serotypes K1 and K2 were the prevalent strains in community-acquired pulmonary infection due to *K. pneumonia*, serotypes were not related to the clinical characteristics and prognosis. The current study found the high prevalence of hypermucoviscosity phenotype as well as the aerobactin and *rmpA *genes in *K. pneumoniae *isolates, which was consistent to the previous investigation of liver abscess strains [16]. The high frequency of coexistence of the aerobactin and *rmpA *genes in the strains causing bacteremic CAP was in agreement with previous reports of liver abscess strains and strains causing bloodstream infections [16,17].

## Conclusions

In conclusion, during 2001-2008, *K. pneumoniae *continued to be the dominant cause of bacteremic CAP in Taiwanese adults in this tertiary-care hospital. The *K. pneumoniae *bacteremic CAP had a higher mortality than *S. pneumoniae *bacteremic CAP. Initial presentation with septic shock and respiratory failure were independent risk factors for both early and total mortality from *K. pneumoniae *bacteremic CAP. Serotypes K1/K2 comprised around half of all isolates, but did not predispose patients to a poor clinical outcome. Physicians should be aware of the poor prognosis of any patient with bacteremic *K. pneumoniae *CAP and monitor these patients closely.

## Abbreviations

CAP: community-acquired pneumonia; 95% CI: 95% confidence interval; OR: odds ratio; APACHE: Acute Physiologic and Chronic Health Evaluation; PSI: pneumonia severity index; ICU: intensive care unit.

## Competing interests

The authors declare that they have no competing interests.

## Authors' contributions

LYT, JYY, and FCP conceived of the study, and participated in its design and coordination. LYT, JYY, and FCP performed the experiments. LYT, JYY, CTL and FCP reviewed the medical records. LYT, JYY, CTL and FCP analyzed and interpreted the data. LYT, JYY, and FCP drafted the manuscript. All authors read and approved the final manuscript.

## Pre-publication history

The pre-publication history for this paper can be accessed here:

http://www.biomedcentral.com/1471-2334/10/307/prepub
